# Aluminum and its potential contribution to Alzheimer's disease (AD)

**DOI:** 10.3389/fnagi.2014.00062

**Published:** 2014-04-08

**Authors:** Surjyadipta Bhattacharjee, Yuhai Zhao, James M. Hill, Maire E. Percy, Walter J. Lukiw

**Affiliations:** ^1^LSU Neuroscience Center, Louisiana State University Health Sciences Center, Louisiana State UniversityNew Orleans, LA, USA; ^2^Department of Ophthalmology, Louisiana State University Health Sciences Center, Louisiana State UniversityNew Orleans, LA, USA; ^3^Department of Microbiology, Louisiana State University Health Sciences Center, Louisiana State UniversityNew Orleans, LA, USA; ^4^Departments of Physiology and Obstetrics and Gynaecology, University of TorontoToronto, ON, Canada; ^5^Neurogenetics Laboratory, Surrey Place CentreToronto, ON, Canada; ^6^Department of Neurology, Louisiana State University Health Sciences CenterNew Orleans, LA, USA

**Keywords:** aluminum, Alzheimer's disease, micro RNA (miRNA), Tg2576, genetic variability, Feralex-G, desferrioxamine

Alzheimer's disease (AD) is perhaps *the principal example* of cognitive failure in humans, and currently over 5.5 million Americans suffer from this incapacitating and progressive disorder of thought, reasoning and memory. Our laboratory has been evaluating the potential contribution of environmentally bioavailable neurotoxic metals to the onset, development and progression of AD for about 30 years (Lukiw et al., 1987). Largely because of its known multiple and potent neurotoxic effects, much of our research has focused on the potential contribution of aluminum to the AD process: (i) because of aluminum's remarkable abundance and bioavailability in the biosphere—in fact it is the most abundant naturally occurring neurotoxic element to which we are exposed; (ii) because of aluminum's remarkable cellular toxicity and genotoxicity at low nanomolar concentrations toward brain genetic processes, and (iii) because of aluminum's highly structured, specific and unique interactions with the phosphate-rich nucleic acids associated with the expression of genetic information in the human brain (Lukiw et al., 1989; Bryant et al., 2004; Alexandrov et al., 2005, 2013; Lukiw and Pogue, 2007; Pogue et al., 2009, 2012; Lukiw, 2010; Percy et al., 2011; Bhattacharjee et al., 2013; De Sole et al., 2013).

Aluminum's contribution to AD is based upon at least seven independently derived observations: (i) that at physiologically realistic concentrations, aluminum strongly promotes amyloid aggregation and accumulation, a key feature of AD neuropathology (Exley, 2005; Rodella et al., 2008; Walton and Wang, 2009; Yumoto et al., 2009); (ii) that both *in vitro* and *in vivo* aluminum promotes inflammatory signaling via the pro-inflammatory transcription factor NF-kB, another prominent feature characteristic of AD brain (Bondy, 2013; Walton, 2013); (iii) that out of the many thousands of brain gene messenger RNA (mRNAs) and micro RNAs (miRNAs), the family of mRNAs and miRNAs induced by aluminum are also strikingly similar to those found to be increased in AD; (iv) that in transgenic animal models of AD dietary aluminum enhances the development of pathological markers such as lipid peroxidation, oxidative stress, apoptosis, and gene expression deficits (Praticò et al., 2002; Bharathi et al., 2008; Zhang et al., 2012); (v) that many of the observed deficits in AD such as chromatin compaction, impaired energy utilization, impaired signaling involving chemical messengers such as adenine triphosphate (ATP) are recapitulated in aluminum-treated cellular or animal models of AD (Alexandrov et al., 2005; Lukiw and Pogue, 2007; Pogue et al., 2012; Bhattacharjee et al., 2013); (vi) that a very significant number of studies link the amount of aluminum in drinking water to the incidence of AD [worldwide, aluminum is added to drinking water as hydrated aluminum potassium sulfate KAl(SO_4_)_2_·12H_2_O, or alum, as a clarification and “finishing” agent (Flaten, 2001; Frisardi et al., 2010; Walton, 2013)], and (vii) perhaps most importantly, that of all pharmaceutical treatment approaches directed against AD to date, chelation using the anti-oxidant and trivalent iron/aluminum chelator desferrioxamine has been shown to be one of the most effective therapeutic strategies yet devised (Crapper McLachlan et al., 1991; Percy et al., 2011).

Abundant research indicates that aluminum is a particularly reactive metal toward multiple aspects of human neurobiology and the altered genetics that are associated with the development and propagation of sporadic AD (Lukiw et al., 1989; Lukiw, 2010; Bhattacharjee et al., 2013; Bondy, 2013; Shaw and Tomljenovic, 2013; Walton, 2013). Thirty years of research since the potent effects of aluminum on the genetic apparatus in AD were first described, the most recent evidence suggests a strong linkage between aluminum sulfates and induction of NF-kB-sensitive pro-inflammatory miRNAs (Lukiw et al., 1987; Alexandrov et al., 2013; Zhao et al., 2013). Aluminum has been previously shown to significantly induce the transcription factor NF-kB (Pogue et al., 2009; Bondy, 2013), and up-regulation of NF-kB drives synthesis of NF-kB-sensitive miRNAs which in turn down regulate the expression of many AD-relevant genes, including complement factor H (CFH) and neurotropic signaling in human brain cells (Pogue et al., 2009; Zhao et al., 2013).

We would like here to briefly include some recent genetic data on aluminum and its effects on miRNA abundance in a highly relevant transgenic animal model for AD that shows strong parallels to miRNA profiles which are found in AD brain (Figure 1). There are currently over 90 transgenic mouse models of AD (http://www.alzforum.org/research-models/). A commonly used Tg2576 mouse model overexpresses a mutant form of beta amyloid precursor protein (β APP), APPK670/671L, linked to early-onset familial AD, and develops amyloid plaques and progressive cognitive deficits as the mice age. Tg2576 mice exposed to dietary aluminum have been shown to develop oxidative stress and robust amyloidogenesis, key features of AD neuropathology (Praticò et al., 2002). Animals provided a 2 mg/kg aluminum-supplemented diet were analyzed for miRNA speciation and complexity in their brains using GeneChip and miRNA array technologies; intriguingly, the same quintet of up-regulated pro-inflammatory miRNAs (miRNA-9, miRNA-34a, miRNA-125b, miRNA-146a, and miRNA-155) as found in (i) AD and (ii) in aluminum-treated human brain cells in primary culture were also found to be amongst the most up-regulated in these aluminum-supplemented Tg2576 mice (Alexandrov et al., 2005; Lukiw and Pogue, 2007; Pogue et al., 2012; Bhattacharjee et al., 2013; Hill et al., 2014; Figure 1; unpublished observations). Up-regulated miRNAs are known to target susceptible mRNAs and down-regulate the expression of many AD-relevant brain genes as is widely observed in AD brain tissues (Colangelo et al., 2002; Guo et al., 2010; Ginsberg et al., 2012). Interestingly, these findings suggest some common miRNA-induced mechanism between two important *in vitro* and *in vivo* models for AD with AD itself. Indeed, the abundance of specific miRNAs are highly selective, and potential indicators and predictors of human health and disease, including progressive neurological disorders such as AD (Alexandrov et al., 2005, 2013; Lukiw and Pogue, 2007; Pogue et al., 2009; Maciotta et al., 2013; Zhao et al., 2013).

**Figure 1:**
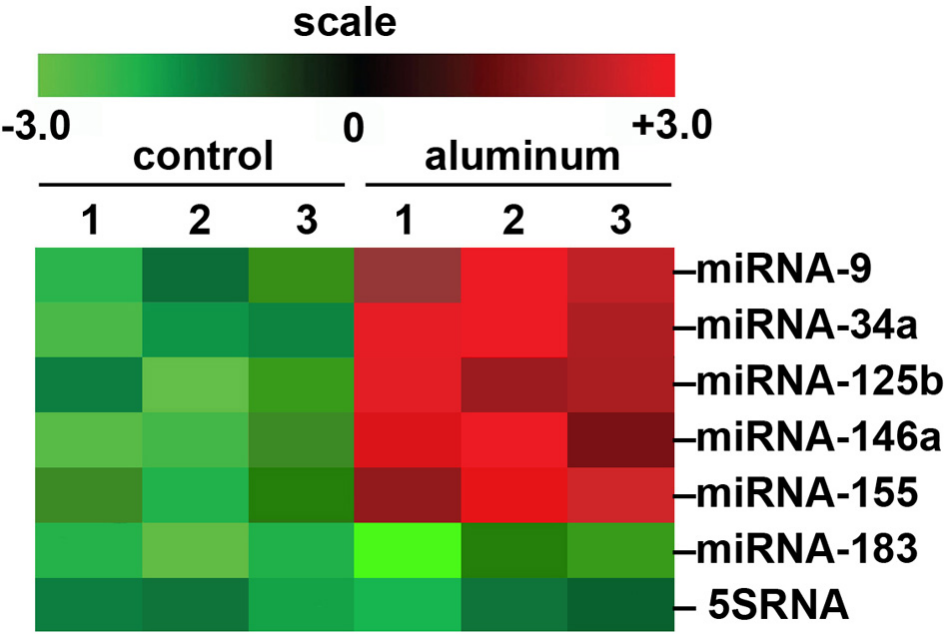
**Array-based cluster analysis of miRNA abundance in aluminum-fed Tg2576 mice vs. controls**. In these experiments the brain (cortex) of 3 month-old Tg2576 mice fed aluminum-enriched diets were analyzed for miRNA speciation compared to age-matched controls (receiving standard diets); methodologies for aluminum-treatment of transgenic animals have been previously described in detail (Praticò et al., 2002; Zhang et al., 2012). Aluminum treatment of other transgenic murine AD models such as the amyloid over-expressing APP/PS1 show similar intensification of AD-type changes (Zhang et al., 2012). The up-regulation of the pro-inflammatory quintet of miRNA-9, miRNA-34a, miRNA-125b, miRNA-146a, and miRNA-155, depicted here, were amongst the most significantly increased miRNAs found to be 2- to 5-fold above normal diet, age-matched controls (compared to an unchanging internal control miRNA-183 and 5SRNA in the same samples). The results strongly suggest a potential contribution of aluminum to the AD processes associated with miRNA-mediated down-regulation of gene expression in the sporadic AD brain as is widely observed (Colangelo et al., 2002; Lukiw and Pogue, 2007; Ginsberg et al., 2012; Pogue et al., 2012; Alexandrov et al., 2013). Importantly, other common environmental neurotoxic divalent metals such as calcium, cadmium, copper, iron (2+), mercury, nickel, and lead, and neurotoxic trivalent metals such as boron, chromium, gadolinium, indium, iron (3+), and yttrium do not exhibit this potentially pathogenic effect (Walker et al., 1989; Alexandrov et al., 2005; Lukiw, 2010; unpublished observations); *N* = 3 control and *N* = 3 aluminum-treated mice; methodologies and data analysis have been extensively described elsewhere (Lukiw and Pogue, 2007; Alexandrov et al., 2013).

Lastly, more research into the potential contribution of aluminum to the AD process is clearly warranted. There are currently no treatments for AD that effectively prevent or cure AD's insidious onset or propagation. We think it important to emphasize that the most effective clinical treatment yet devised for moderate- to late-stage AD patients was the implementation of the first generation anti-oxidant and trivalent iron/aluminum chelator desferrioxamine to attempt to remove aluminum from the brains of AD patients (Crapper McLachlan et al., 1991; Percy et al., 2011). Second generation aluminum chelators such as Feralex-G, either alone or in combination with other chelators, has shown higher specificity, increased selectivity and higher efficacy in aluminum sequestration and chelation in preliminary *in vitro* studies (Kruck et al., 2004, 2008; Percy et al., 2011; unpublished observations). Certainly, laboratory experimentation in cultured primary human brain cells, in transgenic AD animal models, and clinical studies employing next-generation aluminum chelators, perhaps in combination with other drug strategies, are one of the research areas needing more focused attention—to more effectively address the exact role and mechanism of aluminum neurotoxicity in this rapidly expanding healthcare concern.

## Conflict of interest statement

The authors declare that the research was conducted in the absence of any commercial or financial relationships that could be construed as a potential conflict of interest.
